# An Energy-Aware Routing Protocol in Wireless Sensor Networks

**DOI:** 10.3390/s90100445

**Published:** 2009-01-13

**Authors:** Ming Liu, Jiannong Cao, Guihai Chen, Xiaomin Wang

**Affiliations:** 1 School of Computer Science and Engineering, University of Electronic Science and Technology of China, Chengdu, 610054, P.R. China; E-Mail: xiaominwang@163.com; 2 Department of Computing, Hong Kong Polytechnic University, Hong Kong, P.R. China; E-Mail: csjcao@comp.polyu.edu.hk; 3 State Key Laboratory of Novel Software Technology, Nanjing University, Nanjing, 210093, P.R. China; E-Mail: gchen@nju.edu.cn

**Keywords:** Data gathering, Energy-aware, Clustering algorithm, Intra-cluster coverage

## Abstract

The most important issue that must be solved in designing a data gathering algorithm for wireless sensor networks (WSNS) is how to save sensor node energy while meeting the needs of applications/users. In this paper, we propose a novel energy-aware routing protocol (EAP) for a long-lived sensor network. EAP achieves a good performance in terms of lifetime by minimizing energy consumption for in-network communications and balancing the energy load among all the nodes. EAP introduces a new clustering parameter for cluster head election, which can better handle the heterogeneous energy capacities. Furthermore, it also introduces a simple but efficient approach, namely, intra-cluster coverage to cope with the area coverage problem. We use a simple temperature sensing application to evaluate the performance of EAP and results show that our protocol significantly outperforms LEACH and HEED in terms of network lifetime and the amount of data gathered.

## 1. Introduction

With the advances in micro-electro-mechanical system technologies, embedding system technology and wireless communication with low power consumption, it is now possible to produce micro wireless sensors for sensing, wireless communication and information processing. These inexpensive and power-efficient sensor nodes work together to form a network for monitoring the target region. Through the cooperation of sensor nodes, the WSNs collect and send various kinds of message about the monitored environment (e.g. temperature, humidity, etc.) to the sink node, which processes the information and reports it to the user. Wireless sensor networks have a wide-range of applications, including military surveillance, disaster prediction, and environment monitoring, and thus have attracted a lot of attention from researchers in the military, industry and academic fields.

In wireless sensor networks, the sensor node resources are limited in terms of processing capability, wireless bandwidth, battery power and storage space, which distinguishes wireless sensor networks from traditional *ad hoc* networks [15]. In most applications, each sensor node is usually powered by a battery and expected to work for several months to one year without recharging. Such an expectation cannot be achieved without carefully scheduling the energy utilization, especially when sensors are densely deployed (up to 20 nodes/m^3^ [1]), which causes severe problems such as scalability, redundancy, and radio channel contention. Due to the high density, multiple nodes may generate and transmit redundant data about the same event to the sink node, causing unnecessary energy consumption and hence a significant reduction in network lifetime. For a sensor node, energy consumption includes three parts: data sensing, data processing, and data transmission/reception, amongst which, the energy consumed for communication is the most critical. Reducing the amount of communication by eliminating or aggregating redundant sensed data and using the energy-saving link would save large amount of energy, thus prolonging the lifetime of the WSNs.

Data gathering is a typical operation in many WSN applications, and data aggregation in a hierarchical manner is widely used for prolonging network lifetime. Data aggregation can eliminate data redundancy and reduce the communication load. Hierarchical mechanisms (especially clustering algorithms) are helpful to reduce data latency and increase network scalability, and they have been extensively exploited in previous works [2-8]. In this paper, we propose a distributed and energy-efficient protocol, called EAP for data gathering in wireless sensor networks. In EAP, a node with a high ratio of residual energy to the average residual energy of all the neighbor nodes in its cluster range will have a large probability to become the cluster head. This can better handle heterogeneous energy circumstances than existing clustering algorithms which elect the cluster head only based on a node's own residual energy. After the cluster formation phase, EAP constructs a spanning tree over the set of cluster heads. Only the root node of this tree can communicate with the sink node by single-hop communication. Because the energy consumed for all communications in in-network can be computed by the free space model, the energy will be extremely saved and thus leading to sensor network longevity. EAP also utilizes a simple but efficient approach to solve the area coverage problem. With the increase in node density, this approach can guarantee that the network lifetime will be linear with the number of deployed nodes, which significantly outperforms the previous works designed for data gathering application.

The remainder of this paper is organized as follows: Section 2 reviews related works. Section 3 describes the system model and the motivation of our work. Section 4 presents the detailed design of EAP. Section 5 reports the result of EAP effectiveness and performance via simulations and a comparison made with LEACH and HEED. Section 6 concludes the paper.

## 2. Related work

The main task of a sensor network is to forward the sensing data gathered by sensor nodes to the base station. One simple approach to the fulfillment of this task is direct data transmission. In this case, each node in the network directly sends sensing data to the base station. However, if the base station is remote from the sensor node, the node will soon die due to excessive energy consumption for delivering data. To solve this problem, some algorithms aimed at saving energy have been proposed [3-7].

Heinzelman *et al.* [3] proposed an alternative clustering-based algorithm, called LEACH (Low-Energy Adaptive Clustering Hierarchy). It assumes that there exists a unique base station outside the sensor network and all the sensor nodes can communicate with this base station directly. In order to save energy, LEACH only chooses a fraction p of all sensor nodes to serve as cluster heads, where p is a design parameter that must be determined before deployment. The rest sensor nodes join the proper clusters according to the signal strength from cluster heads. In order to share the energy load, its operation is divided into rounds which can guarantee the cluster head rotate in each round. In each round, after cluster formation phase, the cluster heads aggregate the data received from their cluster members and send the aggregated data to the base station by single hop communication, so it can sharply reduce the data needed to be transmitted to the base station.

S. Lindsey *et al.* proposed an algorithm related to LEACH, called PEGASIS [4]. These authors noticed that for a node, within a range of some distance, the energy consumed for receiving or sending circuits is higher than that consumed for amplifying circuits. In order to reduce the energy consumption of sensor nodes, PEGASIS uses the GREED algorithm to form all the sensor nodes in the system into a chain. According to its simulation results, the performance of PEGASIS is better than LEACH, especially when the distance between sensor network and sink node is far large.

In [5], to deal with the heterogenous energy circumstance, the node with the higher energy should have the larger probability to become the cluster head. In this paper, each node must have an estimate of the total energy of all nodes in the network to compute the probability of its becoming a cluster head. As a result, each node will not be able to make a decision to become a cluster head if only its local information is known. In this case, the scalability of this protocol will be influenced.

Sh. Lee *et al.* proposed a new clustering algorithm CODA [6] in order to relieve the inbalance of energy depletion caused by different distances from the sink. CODA divides the whole network into a few groups based on node's distance to the base station and the routing strategy. Each group has its own number of clusters and member nodes. CODA differentiates the number of clusters in terms of the distance to the base station. The farther the distance to the base station, the more clusters are formed in case of single hop with clustering. It shows better performance in terms of the network lifetime and the dissipated energy than those protocols that apply the same probability to the whole network. However, the work of CODA relies on global information of node position, and thus it is not scalable.

Mhatre *et al.* [7] presented a comparative study of homogeneous and heterogeneous networks in terms of overall cost of the network, defined as the sum of the energy cost and the hardware cost. They analyzed both single-hop and multi-hop networks. They used LEACH as a representative homogeneous, single-hop network, and compared LEACH with a heterogeneous single-hop network. The authors indicate that using single-hop communication between sensor nodes and the cluster head may not be the best choice when the propagation loss index k for intra-cluster communication is large (k > 2). They propose a multi-hop version of the LEACH protocol (M-LEACH) and show the cases in which M-LEACH outperforms the single-hop version of the protocol.

In [8], the authors proposed a hybrid, energy-efficient, distributed clustering algorithm (HEED) which periodically selects cluster head according to a hybrid of the node residual energy and a secondary parameter such as node proximity to its neighbors or node degree. Heed terminates in 0 (1) iterations and incurs low message overhead. It achieves fairly uniform cluster head distribution across the network.

The authors in [9] analyze the problem of prolonging the lifetime of a network by determining the optimal cluster size. For a general clustering model, they find the optimal sizes of the cells with which maximum lifetime or minimum energy consumption can be achieved. Based on this result, they propose a location aware hybrid transmission scheme that can further prolong network lifetime.

Besides these clustering algorithms mentioned above, there exist several others algorithms such as those described in [10] and [11]. ACE clusters the network in a constant number of iterations using the node degree as the main parameter. Soro *et al.* [10] proposed an unequal clustering size model for network organization, which can lead to more uniform energy dissipation among cluster head nodes, thus increasing network lifetime. Ye *et al.* [11] proposed a clustering algorithm (EECS) which achieves good cluster head distribution with no iteration and introduces a weighted function for the plain node to make a decision, that which proper cluster should be joined.

## 3. Network Model and Problem Statement

Data gathering application is a typical application in wireless sensor networks. This paper aims at the study of the problems that involve this kind of application. In this section, we will make some assumptions about the network model before problem statement.

### 3.1. Network Model

This paper assumes that N sensor nodes are randomly scattered in a two-dimensional square field A, and the sensor network has the following properties:

1. This network is a static densely deployed network. It means a large number of sensor nodes are densely deployed in a two-dimensional geographic space, forming a network and these nodes do not move any more after deployment.
2. There exists only one base station, which is deployed at a fixed place outside A.
3. The energy of sensor nodes cannot be recharged.
4. Sensor nodes are location-unaware, i.e. a sensor node cannot get its location information through other mechanism such as GPS or position algorithms.
5. The radio power can be controlled, i.e., a node can vary its transmission power depending on the distance to the receiver [5]. For instance, Berkeley Motes [12] have in total 100 power levels.

The first three properties are typical assumptions about wireless sensor networks. These assumptions are widely adopted by most previous works, such as [3 - 6]. For a sensor node, there are three kinds of methods to get its location information i.e. GPS, directional antenna and position algorithms. However, obtaining location information with a hardware device such as GPS or directional antennae will cause an increase in the cost of a sensor node, and position algorithms that need to exchange a large quantity of messages to compute the node's location information will also result in high energy consumption. As a result, the energy cost and system complexity involved in obtaining geography information may compromise the effectiveness of the proposed solutions as a whole. For this reason, the fourth assumption is reasonable. The fifth assumption mainly deals with the definition of different power levels for intra-cluster and inter-cluster communications. In this way, the node energy consumption can be remarkably reduced so as to further prolong sensor network lifetime. In this paper, for simplicity, we assume the power level is continuous.

### 3.2. Problem Statement

There are several requirements for a clustering algorithm. First, a clustering algorithm should be completely distributed because a centralized control manner is not practical in a large-scale sensor network. Second, the cluster heads should be well distributed throughout the monitoring area to make energy consumption be well-balanced among all sensor nodes. Third, the clustering algorithm itself should be energy efficient. Fourth, a clustering algorithm needs to handle the heterogeneous energy circumstance. In reality, it is hard to guarantee that the battery capacity of all nodes is the same. The amount of the energy consumed in gathering data differs among cluster heads, and it depends on the number of cluster members and their positions in the monitoring area. Energy consumption also differs among cluster members due to the different distances to a cluster-head. Furthermore, redeployment for prolonging network lifetime or denser observing will also be the cause that residual energy is not equal among all sensor nodes.

In the current research done in the area of data gathering protocols in wireless sensor networks, we have noticed that most of the proposed clustering algorithms do not satisfy all these requirements. Here, we will make an analysis of three algorithms that may be regarded as representative of the existing research on clustering algorithm in WSNs in order to explain why the proposed clustering algorithms cannot meet all requirements.

LEACH's clustering algorithm assumes that sensor nodes are homogenous and equal. However, in reality, it is hard to guarantee that. In addition, according to the scale of the sensor network, the optimum percentage of cluster heads has to be determined in advance. Therefore, LEACH cannot adapt to such changes in sensor networks as the addition, removal, and transfer of sensor nodes; however, the percentage of cluster heads considerably affects the efficiency of data gathering. Finally, a cluster head needs to broadcast its own advertisement to the whole sensor network in cluster formation phase of LEACH, thus causing another inefficient use of energy.

**Figure 1.**
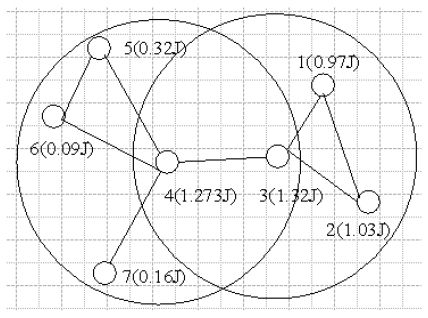
Example for cluster head election.

HEED is a hybrid, energy-efficient, distributed clustering algorithm, but it needs multiple broadcasting for cluster formation and thus consumes more energy. EECS elects cluster heads they should be the nodes with more residual energy in a distributed manner through local radio communication with no iteration while achieving a good cluster head distribution. However, we argue that setting the residual energy as the primarily parameter for cluster heads election doesn't help balance the energy load for the proper nodes, especially in heavy energy heterogeneous circumstance. In most local clustering algorithms in wireless sensor networks, to prolong the sensor network lifetime, the probability of a sensor node's being selected as a cluster head primarily depends on its own residual energy. However, in some special cases, it doesn't help balance the energy load for the proper nodes. As a result, it may cause the problem that some nodes will be exhausted quickly. For instance, as shown in Figure 1, let us consider a sensor network composed by seven nodes.

Nodes 4 and 3 locate in each other's cluster range and the amount of the residual energy of node 4 and node 3 is higher than that of the other nodes. Then, assume that each node's being selected as a cluster head only depends on the residual energy of this node. Obviously, the probability that node 3 is selected as a cluster head is the highest. Consequently, the probability that the other nodes with low residual energy are selected as a cluster head will increase, like node 5 or node 6. Because a cluster head consumes more energy than a plain node, the energy of nodes within the cluster range of node 4 will be exhausted quickly.

In order to solve the problems mentioned above, we present a novel hierarchical clustering scheme EAP which can meet all the requirements listed previously. In the next section, we will describe the EAP algorithm in details.

## 4. Energy-Aware Routing Protocol

Since cluster heads consume more energy than cluster members in receiving sensed data from their member nodes, performing signal processing functions on the data (e.g., data aggregation), and sending the aggregated data to the next hop node or base station, the role of the cluster head must be rotated among all sensor nodes. Therefore, the operation of EAP is divided into rounds as LEACH. Each round begins with a set-up phase while clusters are organized and the routing tree is constructed, followed by a working phase when data are sent to the sink node. For easy reference, we describe the states of nodes and control message in Table 1.

### 4.1. Clustering Algorithm

In EAP protocol, each node needs to maintain a neighborhood table to store the information about its neighbors, as shown in table 2. The ID indicates the unique identification of the neighbor nodes. Without losing the generality, we use an integer value to label a node's identification like TinyOS [14].

At the beginning of each round, each node broadcasts the E_Msg within radio range r and all nodes are cluster head candidates. Here we use r to denote the cluster range. All nodes within the cluster range of one node can be seen as the neighbors of this node. Each node receives the E_Msg from all neighbors in its cluster range and updates the neighborhood table. Using Ea denotes the average residual energy of the cluster range of node V_i_, and V_j_ represents a neighbor node in cluster range of V_i_, where m is the number of nodes within the cluster range. We define:

(1) $$E_{a}=\frac{\sum_{j=1}^{m}V_{j}.E_{\text{residual}}}{m}$$

After exchanging E_Msg, each node computes the broadcasting delay time t for competing cluster head according to the following equation:

(2) $$t=k*T*\frac{E_{a}}{E_{\text{residual}}}$$

where k is a real value uniformly distributed between 0 and 1 and T is the time duration for cluster heads election.

In order to solve the heterogeneous energy problem, EAP uses *E_a_÷ E_residual_* as the primarily clustering parameter for competing cluster heads. Observing equation 2 , t is the time that each node broadcasts the Compete_Msg for competing cluster head, which is mainly determined by *E_a_÷ E_residual_*. As shown in Figure 1, we introduce this clustering parameter for cluster head election. It is easy to find the probability that node 1 becomes a cluster head will increase. This means that the lifetime of the nodes with low residual energy within the cluster range of node 1 will increase. Compared with the previous works [5, 8, 11], which only depend on the residual energy of nodes, EAP can better handle the heterogeneous energy circumstance.

**Figure 2.**
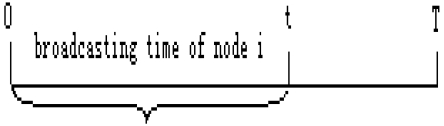
Illustration of broadcasting Compete_Msg.

In EAP, if a node S_i_ has not received any Compete_Msg from its neighbor nodes in the time (0, t), as shown in Figure 2, this node will broadcast the Compete_Msg to all its neighbor nodes. Otherwise, it will give up competition. After S_i_ broadcasts Compete_Msg, it will wait 2* Δ*t*, where Δ*t* denotes the time interval which can guarantee that all neighbor nodes can receive the Compete_Msg, to make sure whether there exists another Compete_Msgs broadcasted by other nodes in its cluster range. If S_i_ has not received any Compete_Msg from its neighbors over Δ*t*, it will set its state as Head, or else it will compare its weight with the weights of other broadcasting neighbors. If S_i_'s weight is the largest one, it will set its state as Head and other broadcasting neighbors give up competition, or else S_i_ sets its state as Plain. Obviously, the CF procedure allows only one cluster head in a cluster range. If there are multiple Compete_Msgs overheard, the one with the largest weight will serve as the only cluster head.

**Figure 3.**
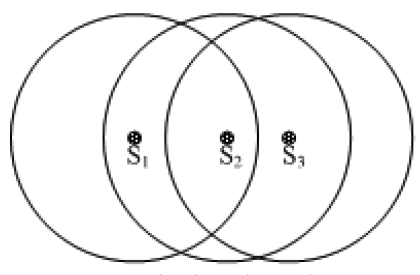
The gradient phenomenon.

It is worth noticing that sometimes there may exist a gradient phenomenon as shown in Figure 3, where *S_1_weight* > *S_2_weight* > *S_3_weight*. Consequently, S2 and S3 will give up competition and S1 is the only winner. In this case, after the clustering phase, some nodes will be neither cluster heads nor member nodes. However, because the time interval Δ*t* is short, the probability that several nodes within the same cluster range broadcast the Compete_Msg in the same time interval (2* Δ*t* is considerably small. In addition, through expanding the time duration T or decreasing the cluster radius, we can guarantee that there is only one head in a cluster range and each node will either be a cluster head or a plain node Whp (with high probability).

**Figure 4.**
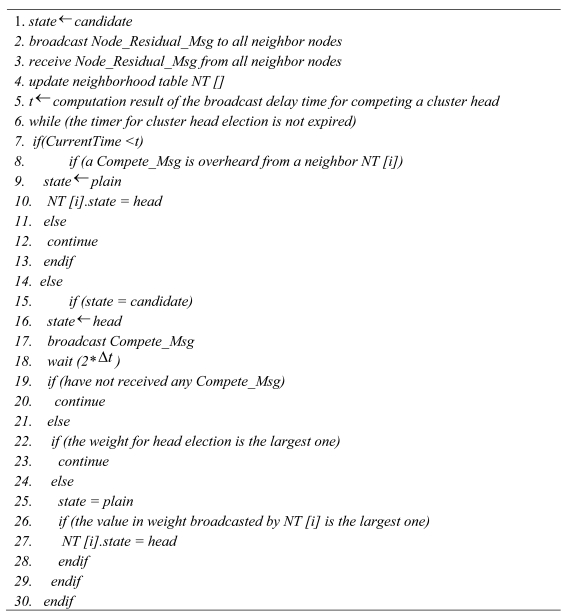
The Pseudo Clustering Algorithm.

In order to minimize the energy consumption in each round, we argue that the plain nodes should join the nearest head. Because the cluster heads always keep rotation in whole lifespan of network, we can maintain uniform energy consumption among all nodes. So minimizing energy consumption for each round can help to prolong the network lifetime. The pseudo code for cluster formation is shown in Figure 4.

### 4.2. Active Member Nodes Selection

Coverage is one of the most important issues in WSNs and it has been studied extensively in recent years [16-18]. In most cases, “coverage” means area coverage. And K-coverage can be described as that every point in the monitored field is covered by at least K sensors. In [16], the authors consider that it is hard to guarantee full coverage for a given randomly deployment area, even if all sensors are on-duty. Small sensing holes are not likely to influence the effectiveness of sensor networks and are acceptable for most application scenarios. It's enough to meet the application's requirements if the active nodes in the network could maintain reasonable area coverage—coverage expectation. Coverage mechanism is to choose a subset of active nodes to maintain the coverage expectation.

We introduce into clusters the notion of “intra-cluster coverage”, which selects some active nodes within clusters while maintaining coverage expectation of the cluster. Utilizing the idea proposed in our research [19], cluster head randomly chooses m' nodes according to equation (3)

(3) $$p_{\text{cover}}=\sum_{i=K}^{m\prime }C_{m\prime }^{i}{\left(\frac{r}{R}\right)}^{2i}{\left(1-\frac{r^{2}}{R^{2}}\right)}^{m\prime -i}$$

where P_cover_ is the coverage expectation of sensing field determined by specific applications; and r is sensing radius, R is cluster radius; m' is the number of active nodes. For example, distributing 200 nodes in a 100×100m^2^ field, r = 12m, R = 30m, then the average number of cluster members is 60 or so. With intra-cluster coverage, if P_cover_ = 99% which means 99% of sensing field is expected to be monitored, 27 members should be active in each cluster to ensure 1-coverage of the cluster and 38 members to ensure 2-coverage. If P_cover_ =95%, only 16 nodes and 25 nodes should be active to ensure 1-coverage and 2-coverage respectively.

Use of intra-cluster coverage has three advantages. The first is to reduce energy consumption in each round by turning redundant nodes' radio off so that network lifetime is prolonged. The second is to reduce TDMA schedule overhead. Once clusters are grouped, all cluster heads broadcast a TDMA schedule packet which contains the members' ID and the slot number allocated to the member. When node density is high, the number of cluster members turns higher so that the length of TDMA schedule packet becomes longer and that means more energy will be consumed to transmit and receive it. However, the length of TDMA schedule packet would not too long with intra-cluster coverage because the number of active node varies slightly when node density goes higher. Apparently, through intra-cluster coverage, EAP can function as a topology control protocol but does not pay any extra energy cost.

### 4.3. Construction of Routing Tree

After the network is clustered, inter-cluster organization depends on the network application. For example, cluster heads can communicate with each other to aggregate their information via multiple hops or communicate with the base station directly. For multi hop communication among cluster heads, the selected transmission range among cluster heads may vary to ensure a certain degree of connectivity and to control interference. For inter-cluster communication, the definition of connectivity depends on its multi hop organization and the relationship between the inter-cluster transmission range, R, the intra-cluster transmission range, r, and the density of nodes. In [8], authors demonstrate that the graph composed by cluster heads will be connected if *R*≥*6r* However, we argue that the theoretical value for connectivity may be not applicable for a real application, i.e. the unreasonable inter-cluster range for inter cluster communication is another inefficient use of energy. For example, we consider a typical setting of sensor network referenced in [8] [network size from (0,0) to (100,100), cluster range = 30 m, sink at (50,175)]. According to the above formulae for connectivity, the radio range for inter-cluster communication should be set as 180 m, which means all cluster heads can almost communicate with base station directly.

In this paper, we set inter-cluster transmission range as 2.5r, where r is the intra-cluster range referenced as before. Because we assume the network is a dense network (> 1/100 m^2^), it can guarantee that most cluster heads are member nodes of the largest connected component of graph composed by all cluster heads. In the next section, we will discuss the relationship between the inter-cluster transmission range, R and the number of independent connected components of a graph by experiments. The theoretical analysis will be made in the later work.

**Figure 5.**
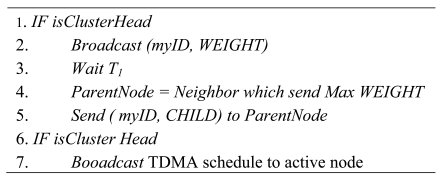
Routing Tree Construction.

After clustering, cluster heads broadcast within a radius R the Weight message, which contains node ID and weight W. The cluster head compares its own weight and the weight contained in the Weight message received from its neighbor cluster head. If it has smaller weight, it selects the node that has the largest weight as its parents and sends the CHILD message to notify the parent node. Finally, after a specified time, a routing tree will be constructed, whose root node has the largest weight among all cluster heads in the same independent connected component. After routing tree construction, cluster heads broadcast a TDMA schedule to their active member nodes to be ready for data gathering. The pseudo code for cluster formation is shown in Figure 5.

**Figure 6.**
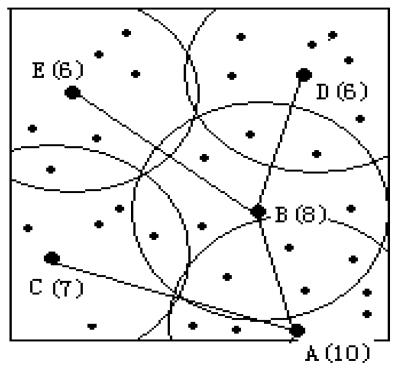
Routing tree construction.

For example, as shown in Figure 6, node A∼E are cluster heads with their weight in parenthesis. B will receive a WEIGHT message from A, C, D, E and select node A to be its parent. Similarly, node D and E choose B as their parent, while C chooses A as its parent. Node A receives a WEIGHT message from nodes B and C, but their weight is less than node A, so A will be the root node that communicates with the base station and routing tree is built.

We define weight *W* of node *i* as:

$$w_{i}=\frac{D\left({\text{RSS}}_{i}\right)\times E_{a}}{D\left({\text{RSS}}_{\max}\right)\times E_{\text{residual}},}$$

where *RSS_i_* denotes node i's received signal strength for the signal broadcasted by the base station, *RSS_max_* is a constant which is determined by the location of the base station, and the function D is used to estimate the distance between node i and the base station. After the deployment of sensors, the base station broadcasts probing message to all sensors and sensors acquire the *RSS* according to the received signal strength. *RSS* remains constant during the network lifetime unless base station varies its location or sensor nodes are mobile. The node that is closer to the base station and locates in a subregion with full energy would be the root node of routing tree due to its higher weight.

## 5. Performance Evaluation

### 5.1. Simulation Parameters

In the simulation experiments, network lifetime has two definitions: First Node Dies (FND), the time when the first node dies in network and Last Node Dies (LND), the time when the last node dies. The parameters of simulations are listed in Table 3. Unless otherwise specified, every simulation result shown below is the average of 200 independent experiments where each experiment uses a different randomly-generated uniform topology of sensor nodes. For simplicity, we assume the probability of signal collision and interference in the wireless channel is ignorable and the radio transmitter, radio amplifier and data fusion unit are the main energy consumers of a sensor node, so we only calculate the energy consumption of these three components in the simulation. In simulation, we use the same radio model shown in [5] for the radio hardware energy dissipation. This radio model has been widely adopted in several studies [3, 6, 10-11].

### 5.2. Simulation results

**Figure 7.**
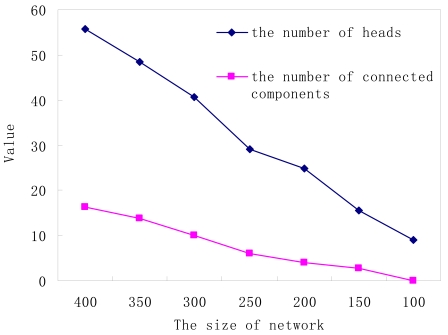
The number of heads and the number of connected components.

Figure 7 shows the relationship between the number of cluster heads, the number of independent connected components, and network size. As the network size increases, it can be seen that the number of independent components is increasing too.

When the network size is (100 × 100), the number of connected components equals 1 in most cases, which means the graph composed of cluster heads is connected. When the network size is (400 × 400), after the clustering phase, almost sixty cluster heads will be generated. However, such a large number of nodes cannot guarantee the connectivity of all cluster heads. As shown in Figure 7, the number of independent components almost reaches 20. Obviously, our algorithm can work well when the node density is high enough, that is, more than 0.01/m^2^.

**Figure 8.**
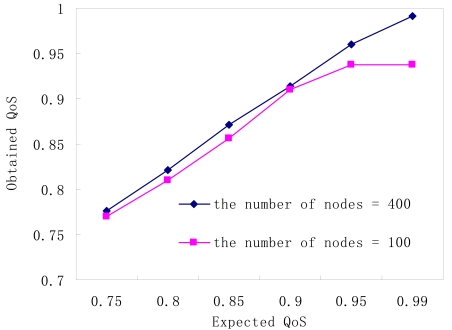
Expected QoS vs. obtained QoS.

Figure 8 proves that EAP can effectively provide the required QoS. On the one hand, providing QoS lower than the required one may save energy at a risk of failing to meet the application's requirement. On the other hand, providing a higher QoS than required by a specific application will decrease the efficiency of energy utilization. However, EAP cannot provide a perfect matching between expected QoS and obtained QoS. It maybe does not work well in some applications, which strictly require that the deviation between expected QoS and obtained QoS is small enough. As shown in Figure 8, if there are 100 nodes deployed in monitoring area, the obtained QoS cannot meet the application requirement when the expected QoS exceeds 95%, because even if all nodes are turned on, they cannot cover a 95% fraction of the whole monitoring area.

**Figure 9.**
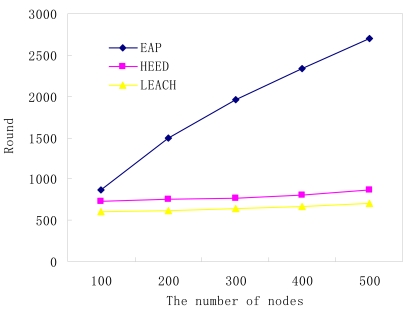
The number of nodes vs. network lifetime (expected QoS = 0.95).

**Figure 10.**
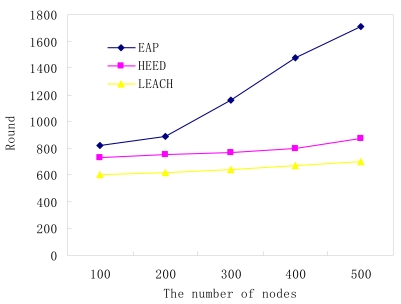
The number of nodes vs. network lifetime (expected QoS = 0.99).

Because HEED and LEACH cannot provide the topology control function, each node needs to collect the temperature information and transmit it to its cluster head, even if it is a redundant node. So, the two algorithms fail to prolong network lifetime when node density is high. Conversely, for the EAP protocol, through the intra-cluster coverage method, the number of actual active nodes is only determined by the expected QoS. As shown in Figures 9 and 10, it is easy to find that the sensor network lifetime will be almost be linear in the number of nodes which are deployed in monitoring area.

**Figure 11.**
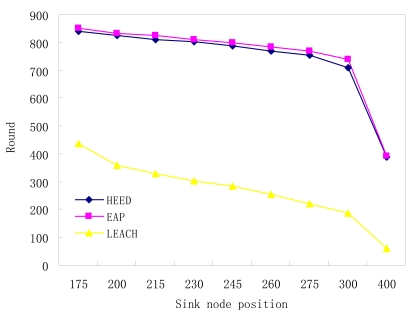
Sink node position vs. network lifetime.

Assume that there are 100 nodes distributed in a monitored region which area is 100× 100 m2, where the sink node's position ranges from (50, 175) to (50, 400) and the cluster range (for intra cluster communications) in EAP and HEED is set to 30m. The monitored region requires being a hundred per cent covered, i.e. all the nodes are working nodes. The stimulation results are shown in Figure 11. With the increase of the distance between the sink node and the network, the energy consumption of the nodes that can directly communicate with the sink node will increase remarkably. In this case, the larger the number of the nodes that can directly communicate with the sink node is, the more rapidly the performance of protocols degenerates. Therefore, EAP and HEED, in which protocols there is only a single node to communicate with the sink node, perform remarkably better than LEACH. In LEACH, all the nodes should take turns to be a cluster head to communicate with the sink node, and since the distance between each node and the sink node is different, the energy consumption for each node is different. As a result, some node with higher energy consumption will die soon. As Figure 11 shows, the network lifetime of EAP and HEED is over 200 rounds longer than that of LEACH.

## 6. Conclusions

In this paper, we present EAP, a novel energy efficient data gathering protocol with intra-cluster coverage. EAP clusters sensor nodes into groups and builds routing tree among cluster heads for energy saving communication. In addition, EAP introduces the idea of area coverage to reduce the number of working nodes within cluster in order to prolong network lifetime. Simulation results show EAP outperforms far better than LEACH. Compared to HEED, though EAP performs almost the same as HEED when node density is low, it has far better performance than HEED when node density goes higher than 0.01nodes/m^2^.

## Figures and Tables

**Table 1.** Descriptions of States and Messages.

**State or Message**	**Description ^a^**
Candidate	The node is a candidate node
Head	The node is selected as cluster head
Plain	The node is a member node
Compete_Msg	Composed by the ID of sender
Join_Msg	Composed by the ID of sender and the ID of head
Weight_Msg	Composed by the ID of sender, weight, Ea, Eresidual
Schedule_Msg	Head assign slot time for its member nodes

**Table 2.** Node 4's Neighborhood Table.

**ID**	**State**	**Residual Energy (J)^a^**
3	Candidate	1.32J
7	Candidate	0.16J
6	Candidate	0.09J
5	Candidate	0.32J

**Table 3.** Simulation Parameters.

**Parameters**	**Value**
Network Filed	*(0,0)∼(100,100)*
Node numbers	*100∼500*
Cluster radius r	*30 m*
Sensing radius rs	*10 m*
Sink position	*(50,200)*
Initial energy	*2 J*
Data packet size	*525 Bytes*
Broadcast packet size	*25 Bytes*
Ethreshold	*0.01 J*
Eelec	*50 nJ/bit*
efs	*10 nJ/bit/m^2^*
eamp	*0.0013 pJ/bit/m^4^*
EDA	*5 nJ/bit/signal*
Threshold distance d0	*75 m*
Data Cycles per round(L)	*5*
